# 

**Published:** 1891-11-07

**Authors:** 


					rHOMAS'SWsS"i
.A-saair Wcs teah Infirmary
.amuHO&W/CH HOS_PLTAM
WITH EXTRA CURSING SUPPLEMENT.
No. 267. Vol. XI.] Saturday,
November 7th, 1891.
[One PENNi.

				

